# Postnatal stem/progenitor cells derived from the dental pulp of adult chimpanzee

**DOI:** 10.1186/1471-2121-9-20

**Published:** 2008-04-22

**Authors:** Pei-Hsun Cheng, Brooke Snyder, Dimitri Fillos, Chris C Ibegbu, Anderson Hsien-Cheng Huang, Anthony WS Chan

**Affiliations:** 1 Neuroscience Division, Yerkes National Primate Research Center, Atlanta, USA; 2 Department of Human Genetics, Emory University School of Medicine, Atlanta, USA; 3 Department of Microbiology and Immunology, Emory University School of Medicine, Atlanta, USA; 4 Emory Vaccine Center, Yerkes National Primate Research Center, Atlanta, USA; 5 Genetics and Molecular Biology Program, Emory University School of Medicine, Atlanta, USA; 6 Neuroscience Program, Emory University School of Medicine, Atlanta, USA; 7 Grace Dental Clinic, Kaohsiung City, Taiwan, PRoC

## Abstract

**Background:**

Chimpanzee dental pulp stem/stromal cells (ChDPSCs) are very similar to human bone marrow derived mesenchymal stem/stromal cells (hBMSCs) as demonstrated by the expression pattern of cell surface markers and their multipotent differentiation capability.

**Results:**

ChDPSCs were isolated from an incisor and a canine of a forty-seven year old female chimpanzee. A homogenous population of ChDPSCs was established in early culture at a high proliferation rate and verified by the expression pattern of thirteen cell surface markers. The ChDPSCs are multipotent and were capable of differentiating into osteogenic, adipogenic and chondrogenic lineages under appropriate *in vitro *culture conditions. ChDPSCs also express stem cell (Sox-2, Nanog, Rex-1, Oct-4) and osteogenic (Osteonectin, osteocalcin, osteopontin) markers, which is comparable to reported results of rhesus monkey BMSCs (rBMSCs), hBMSCs and hDPSCs. Although ChDPSCs vigorously proliferated during the initial phase and gradually decreased in subsequent passages, the telomere length indicated that telomerase activity was not significantly reduced.

**Conclusion:**

These results demonstrate that ChDPSCs can be efficiently isolated from post-mortem teeth of adult chimpanzees and are multipotent. Due to the almost identical genome composition of humans and chimpanzees, there is an emergent need for defining the new role of chimpanzee modeling in comparative medicine. Teeth are easy to recover at necropsy and easy to preserve prior to the retrieval of dental pulp for stem/stromal cells isolation. Therefore, the establishment of ChDPSCs would preserve and maximize the applications of such a unique and invaluable animal model, and could advance the understanding of cellular functions and differentiation control of adult stem cells in higher primates.

## Background

Human DPSCs (hDPSCs) were first reported in 2000 [1] and were categorized as post-natal stem cells. DPSCs are capable of differentiating into osteogenic lineages with functions similar to those of osteoblasts [1-3]. A recent study demonstrated the expression of bone markers in DPSCs, including bone sialoprotein, alkaline phosphatase, osteocalcin, and type-I collagen [1]. In addition, like osteoblasts, hDPSCs can differentiate into adipocytes [1,2]. Although the cell therapeutic potential of DPSCs has not been fully investigated, additional evidence of the multipotent differentiation potential of DPSCs has strongly suggested that DPSCs are able to differentiate beyond mineralized tissues [1,4-6].

The differentiation capacity and multipotency of DPSCs could be greatly enhanced by the selection of a subset of DPSCs based on cell surface marker expression and DNA dyes exclusion [4,6]. DPSCs and BMSCs have similar gene expression profiles, although their ability to differentiate varies [7]. Recent discoveries suggest that BMSCs are capable of stimulating neurogenesis through the release of growth factors and the induction of local repair responses [8,9]. Furthermore, BMSCs have also been considered to be a potential treatment for tumors [10,11], neurodegenerative diseases [12,13], and diabetes mellitus [14]. Although there is evidence supporting the notion that BMSCs have potential therapeutic applications, it is yet to be determined whether DPSCs also have a therapeutic function.

In addition to their 98% genetic similarity to humans [15,16], chimpanzees also show similar cognitive and behavioral characteristics [17-21]. These similarities are sufficient to suggest a critical role for the chimpanzee modeling of human medical conditions, such as Autoimmune Deficiency Syndrome (AIDS), Alzheimer's disease (AD), cancer, malaria, and perimenopausal complications [16,22-24]. Although mouse, rat, fish and other animal models for these conditions have been developed, the differences in physiology, genetics and pathology from humans make it difficult to extrapolate findings to significant breakthroughs in human medicine [16,25-27]. Due to the unique genetic and physiologic homology of chimpanzees to humans [16,28], chimpanzees have been widely used in studies of hepatitis B and C viruses [29-33], AIDS [34,35], reproduction [36-38] and behavioral development [18-20,39-41]. However, a chimpanzee model for stem cell research has yet to be developed.

Stem cell research in chimpanzees has not been at the forefront of the field due to limited access, limited availability and highly restricted usage of chimpanzees in research. As delineated above, the unique characteristics of chimpanzees, including a high genomic proximity and close pylogenetic relation with humans, indicate that chimpanzee stem cells could lead to insights into the regulatory mechanisms of gene regulation and differentiation control in humans. There has been much interest in embryonic stem (ES) cells, which have a well-defined differentiation capacity; human, monkey and rodent models have provided an enormous amount of information for comparative studies. However, the establishment of chimpanzee ES cells is not an easy task. DPSCs are easier to obtain than ES cells, however there is much skepticism as to whether or not DPSCs are true pluripotent cells that can mature into fully functional cells from each germ layer [1,4-6]. Similar to BMSCs, DPSCs are considered as a subgroup of MSCs with different differentiation capability. Although BMSCs could also be recovered during necropsy, DPSCs are alternative source of MSCs with distinctive features that make it a unique and valuable comparative model for elucidating the origin and characteristics of MSCs and ASCs. The recent changes in chimpanzee policy have further reduced the accessibility of chimpanzees in biomedical research. Unlike other animal models, such as monkeys and mice, chimpanzees provide a unique model for the potential discovery of minute differences resulting from the speciation of gene function in humans [23]. Thus, the scientific role of chimpanzees in biomedicine needs to be redefined, and a new venue of chimpanzee modeling is necessary in order to preserve this unique and important model for comparative medicine.

The present study is intended to develop an alternative strategy to preserve and maximize the potential applications of a chimpanzee model for adult stem cell research. Our goal is to evaluate the feasibility of establishing ChDPSCs from post-mortem tissues and determine their multipotent differentiation capacity through direct comparison with hBMSCs and cell surface antigen profiling. We aim to develop ChDPSCs and determine if they can be used as an alternative source of stem cells and serve a novel comparative model in adult stem cell research, which may lead to an increased understanding of stem cell properties and differentiation control in higher primates.

## Methods

### Isolation and culture of ChDPSCs

An incisor and a canine tooth of a forty-seven year old female chimpanzee, euthanized for clinical reasons at the Yerkes National Primate Research Center, were recovered during necropsy. The teeth were stored in DPBS on ice, and delivered to the laboratory. The teeth surfaces were cleaned and dissected at the cementum-enamel junction to reveal the pulp chamber. The dental pulp was recovered followed by digestion in 3 mg/ml collagenase type I (Invitrogen, Inc.) and 4 mg/ml of dispase (Invitrogen, Inc) for one hour at 37°C [1]. The cell suspension was filtered through a 70 μm cell strainer (Falcon, Inc) to recover the single-cell suspensions. The single-cell suspensions of the dental pulp were then cultured in a DPSC medium (α-MEM) supplemented with 20% fetal bovine serum (Hyclone, Inc), 100 μm L-ascorbic acid-2-phosphate (Sigma, Inc), 2 mM glutamine, 100 units/ml penicillin and 100 μg/ml streptomycin (Invitrogen, Inc.), and incubated at 37°C with 5%CO_2_. Medium was replaced every three to four days and cells were passaged at 70% confluence.

### Culture of hBMSCs

hBMSCs were acquired from the Tulane University Health Sciences Center. In brief, hBMSCs were cultured in α-MEM (GIBCO/BRL, Grand Island, NY) supplemented with 20% fetal bovine serum (Atlanta Biologicals, Inc), 100 units/ml penicillin (Invitrogen, Inc.), 100 μg/ml streptomycin (Invitrogen, Inc.), and 2 mM L-glutamine (Invitrogen, Inc.). Cells were then cultured at 37°C with 5% humidified CO_2_. The hBMSCs were passaged and maintained at low density. hBMSCs were re-plated at 50 cells/cm^2 ^and were harvested at 70% confluence.

### Proliferation rate of ChDPSCs

The proliferation rate of ChDPSCs was determined by cell count and MTT assay (ATCC), which was performed every five passages (P5, P10 and P15). For cell count: A total of 5 × 10^4 ^cells were plated in a 35 mm dish and a total of three replicas for each cell line were prepared at P5, P10 and P15. At 96 hours post-seeding, ChDPSCs were harvested and a total cell count was determined. For MTT cell proliferation assay: Cell proliferation was determined by using the ATCC MTT Cell Proliferation Assay Kit. A total of 5% or 10% of the ChDPSCs sample from the cell count study was seeded in triplicates in a 96 well plate and cultured overnight. A serially diluted 293FT cell of known number was plated in the same 96 well plate in order to generate a standard curve for later normalization. Each well was brought up to 100 μl total volume and cultured overnight. On the following day, all ChDPSC wells were replaced with 100 μl of fresh culture medium and 10 μl of the MTT reagent was added to each sample and the blank control. After four hours incubation, 100 μl of detergent was added to each well, the plate was covered and kept in the dark at room temperature overnight. Plates were measured with an ELISA reader using an absorbance wavelength of 570 nm on the following day. The readings of the ChDPSCs were normalized by a standard curve based on the 293FT cell and compared between different passages.

### Telomere length assay

To determine telomere length, the TeloTAGGG Telomere Length Assay kit from Roche Applied Science was used. In brief, genomic DNA was digested with a mixture of *Hinf I *and *Rsa I *restriction enzymes. Digested DNA was then separated in 0.8% agarose gel followed by depurination, denaturation, and neutralization, transferred onto a positively charged nylon membrane. The membrane was hybridized with a DIG labeled telomere probe at 42°C. Three hours later, the membrane was washed in a high stringency buffer and incubated with Anti-DIG-AP solution. After the final wash, AP substrate was applied and exposed on X-ray film.

### Colony forming unit (CFU)

Colony forming unit was determined by plating 30 cells/35 mm culture dish followed by 2 weeks culture in a DPSC medium. Medium was replaced every three to four days. After 14 days in culture, the cells were fixed in 100% methanol for 20 minutes followed by 3% crystal violet staining. Any colonies larger than 2 mm in diameter were counted. The CFU equals the total number of colonies divided by the initial number of cells multiplied by 100 as a percentage.

### Adipogenic, osteogenic and chondrogenic differentiation

*For adipogenic differentiation*, the cells were seeded at 400 cells per 35 mm tissue culture dish and cultured for 11 days in DPSC medium. On day 11, the DPSC medium was supplemented with 5.0 μg/ml insulin, 50 μM indomethacin, 1 μM dexamethasone, and 0.5 μM IBMX, which was subsequently replaced every three to four days for three weeks. The culture was then fixed in 4% paraformaldehyde (PFA) followed by Oil-Red-O stain.

*For osteogenic differentiation*, the cells were prepared as described for adipogenic differentiation until day 11. On day 11, the DPSC medium was supplemented with 1 nM dexamethasone, 50 uM L-Ascorbic acid 2-phosphate sesquimagnesium salt, 20 mM β-glycerolphosphate, and 50 ng/ml L-thyroxine sodium pentahydrate, and was subsequently replaced every three to four days for three weeks. The culture was then fixed in 4% PFA followed by Alizarin Red S stain.

*For chondrogenic differentiation*, 2.5 × 10^5 ^ChDPSCs were centrifuged in a 15 ml conical tube at 1,000 rpm for five minutes. The pellet was maintained in a DPSC medium supplemented with ITS-plus premix (BD Biosciences) to a final concentration of 6.25 ug/ml insulin, 6.25 ug/ml transferrin, and 6.25 ng/ml selenious acid. Additionally, 5.35 ug/ml linoleic acid, 1.25 mg/ml bovine serum albumin, 50 μg/ml Ascorbate 2-phosphate, 40 μg/ml L-proline, 100 μg/ml Sodium pyruvate, 100 nM Dexamethasone, 100 units/ml penicillin, 100 μg/ml streptomycin and 10 ng/ml TGF-β3 (R&D Systems) were also supplemented. Medium was replaced every three to four days for four weeks. The pellets were then fixed in 4% PFA overnight, and the paraffin-embedded sections (4–5 μm) were stained with Alcian blue.

#### RT-PCR

RNA from cell samples of ChDPSCs were prepared with RNeasy Mini kit (Qiagen) for RNA purification and RNase-Free DNase set (Qiagen) for the removal of genomic DNA in the RNA sample. 1.5 ug of total RNA were used to synthesize cDNA by SuperScript III Reverse transcriptase (invitrogen). PCR was performed using a specific primer set targeting stem cell markers: Oct 4 (Oct4-F: 5'- GCA ACC TGG AGA ATT TGT TCC T-3' and Oct4-R: 5'- AGA CCC AGC AGC CTC AAA ATC -3' with an amplicon of 182 bp), Rex-1 (Rex1-F: 5'- GTG AAC AGA ACA GAA GAG GCC -3' and Rex1-R: 5'- GAA ATC GTC CTC TCC AAC AGC -3' with an amplicon of 350 bp), Sox-2 (Sox2-F: 5'- GCA GGT TGA CATC GTT GGT AAT -3' and Sox2-R: 5'- CAA CTA CGG AAA ATA AAG GGG G -3' with an amplicon of 179 bp) and Nanog (Nanog-F: 5'- TCT CTC CTC TTC CTT CCT CCA -3' and Nanog-R: 5'- GGA AGA GTA GAG GCT GGG GT -3' with an amplicon of 389 bp), and differentiation markers: osteonectin (osteonectin-F: 5'- ATC TTC TTT CTC CTT TGC CTG G -3' and osteonectin-R: 5'- GCA CAC CTC TCA AAC TCG CC -3' with an amplicon of 323 bp), osteocalcin (osteocalcin-F: 5'- AGG TGC GAA GCC CAG CGG T -3' and osteocalcin-R: 5'- GCC AGC AGA GCG ACA CCC T -3' with an amplicon of 258 bp), osteopontin (osteopontin-F: 5'- CAG TGA TTT GCT TTT GCC TCC T -3' and osteopontin-R: 5'- CAT TCA ACT CCT CGC TTT CCA T -3' with an amplicon of 507 bp). Bone sialoprotein (BSL-F: 5'- CAC TGG GCT ATG GAG AGG AC -3' and BSL-R: 5'- GCC CTT GCC CTG CCC TCC -3' with an amplicon of 338 bp). GAPDH (GAPDH-F: 5'- ATG GGG AAG GTG AAG GTC GG -3' and GAPDH-R: 5'- CCA TCA CGC CAC AGT TTC CC -3' with an amplicon of 596 bp).

#### Flow cytometry analysis

Cell lines were divided into 15 FACS tubes (BD Biosciences) at 2 × 10^5 ^cells/tube and stained with FITC or PE-conjugated anti-CD14, -CD45, -CD59, -CD73, -CD90, -CD150, -CD166, -IgG1k, -IgG2ak (all from BD Pharmingen), or anti-CD18, -CD24, -CD29, -CD34, -CD44 (all from BD Biosciences), or anti-CD105 (eBioscience). After incubating 20 minutes at ambient temperature in the dark, cells were washed with 2 mL FACS wash solution (dPBS+1%BSA+0.1%NaN_3_) and centrifuged 5 minutes at 230 × g. Supernatant was removed and cells were fixed with 1% formaldehyde (in dPBS). All data was acquired using a FACS Calibur (Becton Dickinson) and analyzed using CellQuest (Becton Dickinson) and Flowjo software (Treestar, Inc.).

#### Statistical Analysis

Data analyses were carried out by *Student *t-test.

## Results

### Isolation and proliferation rate of ChDPSCs

**Figure 1:**
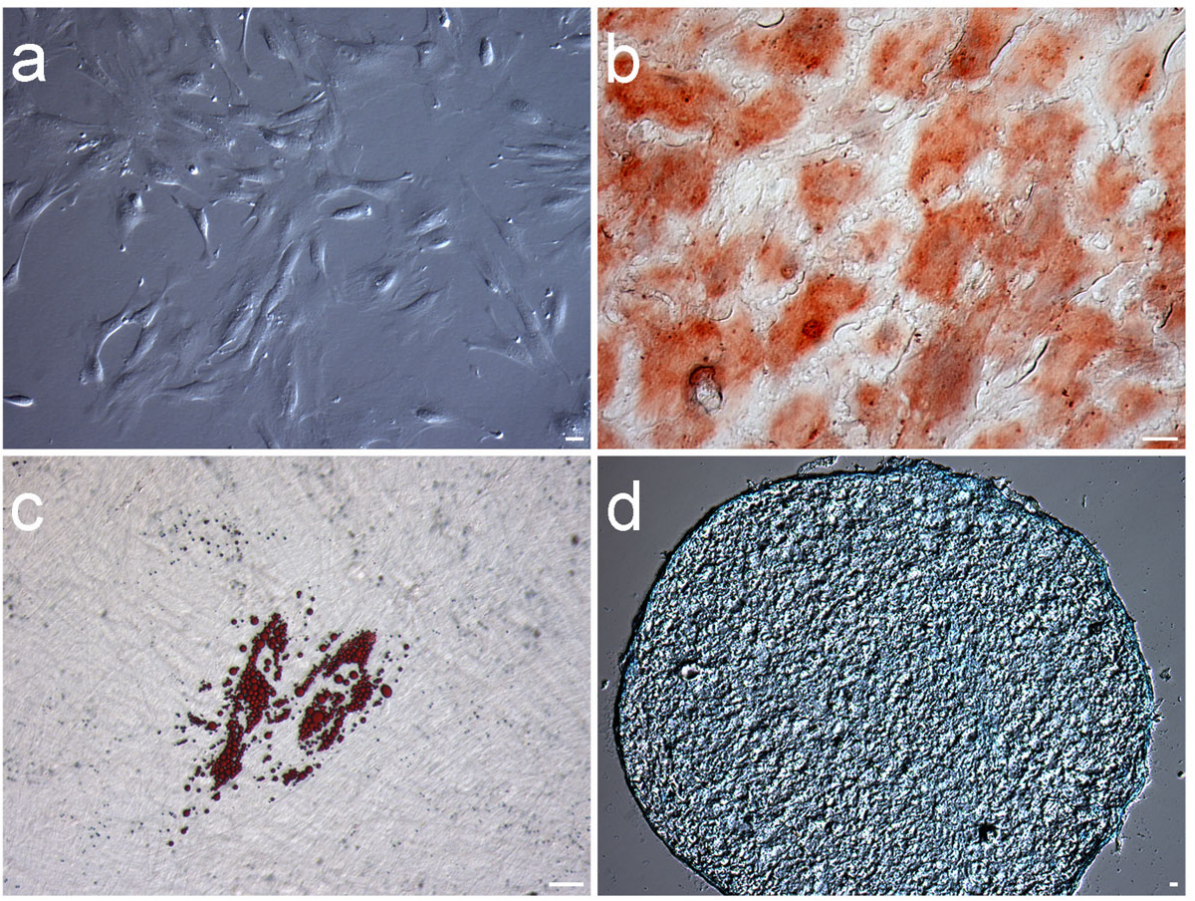
**Isolation, culture and differentiation of ChDPSCs.** (a) Spindle and fibroblast-like cells, similar to those of the hBMSCs, were observed in ChDPSC culture. (b) Osteogenic and (c) Adipogenic differentiation were demonstrated by three weeks induction followed by Alizarin Red S and Oil-Red O staining, respectively. (d) Chondrogenic differentiation was demonstrated by four weeks induction followed by Alcian blue staining of cryosection. Bar = 5 μm.

**Figure 2:**
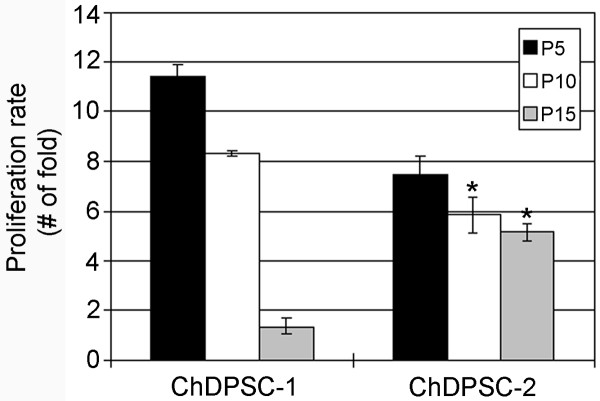
**Proliferation of ChDPSCs.** The proliferation rate of ChDPSC-1 and ChDPSC-2 was determined by the total cell number at every five passages until passage 15 while an equal number of cells were seeded and cultured for 96 hours before cell counting. Columns with an asterisk indicates no significant difference (P > 0.05).

DPSCs have previously been isolated from humans and other species, including mice and pigs [1,2,4]. Here we reported the isolation of ChDPSCs from post-mortem teeth of a forty-seven year old female chimpanzee. The chimpanzee is one of the closest relatives to humans, and ChDPSCs could serve as a powerful comparative model for the study of stemness and differentiation control of adult stem cells in higher primates. The teeth were retrieved from a chimpanzee euthanized at the Yerkes National Primate Research Center (YNPRC), acquired through the tissue distribution program. ChDPSCs share similar morphology to hBMSCs, which are long and spindle shaped (Figure 1a). Two ChDPSC lines were established from the dental pulp of an incisor (ChDPSC-1) and a canine (ChDPSC-2). Both cell lines were capable of forming colonies, with a colony forming unit of 33% and 31% for ChDPSC-1 and ChDPSC-2, respectively, which suggests their capacity for self-renewal and propagation. ChDPSCs have a high proliferation rate at initial and early passages, which decreases gradually in culture (Figure 2). ChDPSC-1 had a higher proliferation rate (11.43 ± 0.47 folds) at passage five compared to that of ChDPSC-2 (7.52 ± 0.69 folds). However, the growth rate of ChDPSC-1 decreased significantly by passage 15 (1.37 ± 0.3 folds) whereas the growth rate of ChDPSC-2 (5.13 ± 0.35 folds) reduced more slowly (p < 0.05; Figure 2). The proliferation study was conducted using two different methods, cell count and MTT proliferation assay, which revealed consistent results.

### Assessment of telomerase length in ChDPSCs

**Figure 3:**
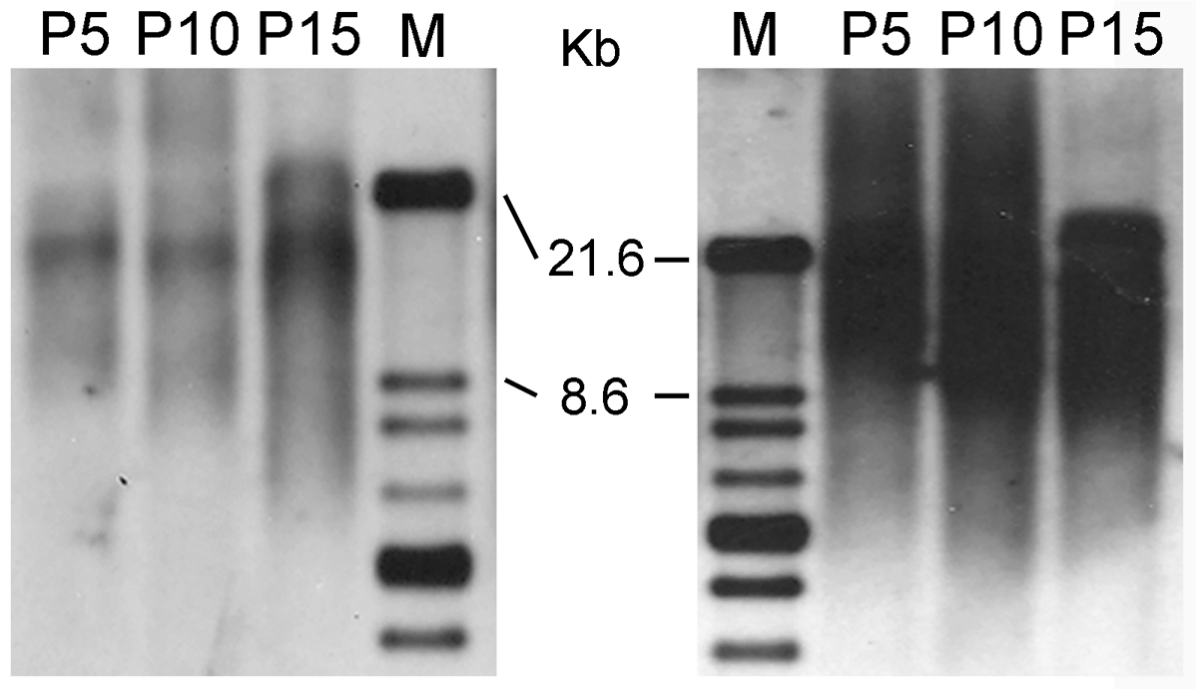
**Telomere length analysis of ChDPSC-1 and ChDPSC-2.** Telomere length of ChDPSCs was measured by using the TeloTAGGG Telomere Length Assay kit from Roche Applied Science. Cell samples were collected at every five passages until passage 15 as described in the proliferation study. A reduced telomere length was observed in both ChDPSC lines as the number of passage increase. Left panel: ChDPSC-1; Right panel: ChDPSC-2.

Telomere length is one of the most commonly used markers in aging studies. Telomere length decreases as cells continue to divide *in vitro *and is a natural process in aging [42]. In general, long telomeres (10–20 kb) are observed in stem cells or cells that are vigorously proliferated [43], whereas the length of telomeres in somatic cells is shorter and ranges from 5–15 kb [44]. To determine the length of the telomeres, a commercially prepared assay was used. Longer telomeres were observed in earlier passages (P5), and while the telomere length gradually decreased in later passages (Figure 3), most of the telomeres remained between 10–20 kb in length. This result is consistent with the results of a prior proliferation study, in which a reduced growth rate of ChDPSCs in culture was parallel with a decrease in telomere length.

### Expression of stem cell and differentiation markers in ChDPSCs

**Figure 4:**
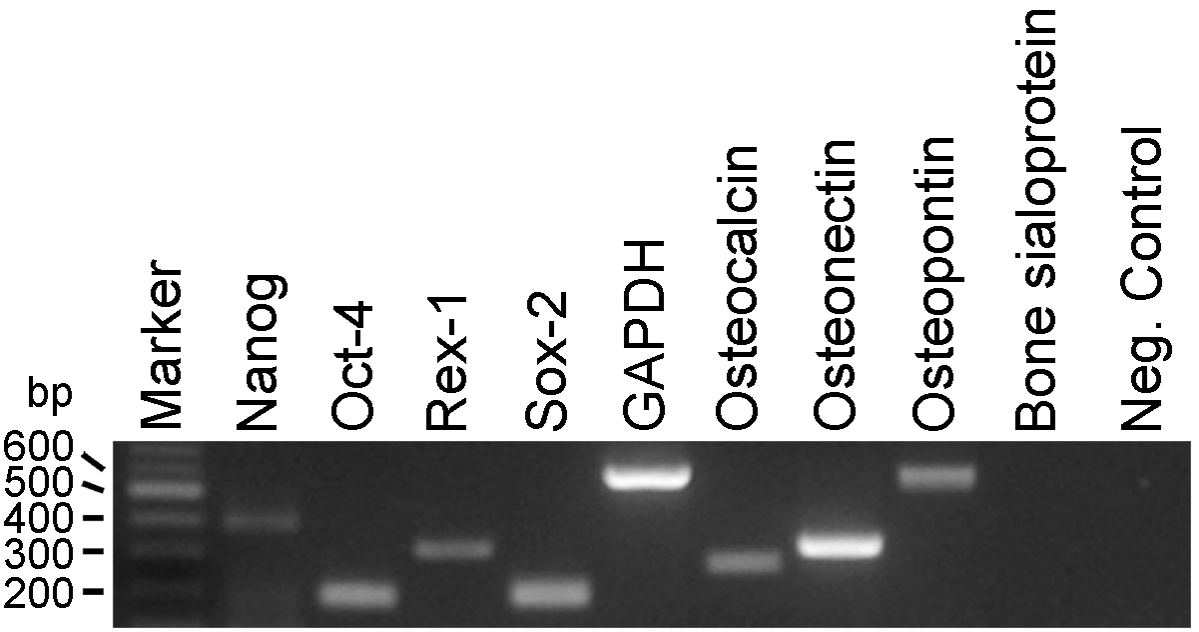
**Expression of stem cell markers.** mRNA isolated from ChDPSCs was used for RT-PCR analysis. The expression of stem cell (Nanog, Rex-1, Oct-4) and differentiation (Osteonectin, Osteocalcin, Osteopontin) markers was detected. Bone sialoprotein was not detected in ChDPSCs. Internal control: GAPDH. Negative control: water. All primers were designed to cross the junction between two exons with the upstream primer located at the 3'region of the leading exon; whereas, the down stream primer was located at the 5'region of the following exon in order to minimize the possibility of a false positive or contamination of genomic DNA.

In order to determine the multipotent differentiation capability of ChDPSCs, the expression of stem cell specific transcription factors was determined. In addition to stem cell markers, the expression of differentiation markers, such as osteonectin, osteocalcin, osteopontin which is expressed in precursor osteoblasts, was also determined. The expression of the stem cell (Nanog, Sox-2, Rex-1 and Oct-4) and differentiation (Osteonectin, osteocalcin, osteopontin) markers was confirmed in both ChDPSC lines (Figure 4) by RT-PCR analysis using specific primer sets. A similar expression pattern was also reported in rBMSCs [42] and hDPSCs [1,2]. Our results suggested the stem cell properties of the ChDPSCs, which were further confirmed in the *in vitro *differentiation study.

### Osteogenic, adipogenic and chondrogenic differentiation of ChDPSCs

Two of the main characteristics of stem cells are self-renewal or self-propagation and competency for multipotent differentiation. Differentiation into osteogenic, adipogenic and chondrogenic lineages is considered to be a trademark event of BMSCs [45] and DPSCs [1,2,7]. Both ChDPSC lines demonstrated potential for osteogenic, adipogenic and chondrogenic differentiation. The osteogenic capability of ChDPSCs (Figure 1b) was comparable to that of hBMSCs [45], hDPSCs [2] and rBMSCs [42]. Although the adipogenic capability of hDPSCs [1,2,7] has not been clearly demonstrated, adipogenic differentiation of ChDPSCs was clearly confirmed by the formation of fat droplets (Figure 1c), which were morphologically comparable to that of hBMSCs. Furthermore, ChDPSCs were also capable of differentiating into chondrogenic lineages (Figure 1d). Here, we have confirmed the multipotent differentiation capability of ChDPSCs and that this capability is comparable to that of hBMSCs and hDPSCs.

### Cell surface antigen profile in ChDPSCs

**Figure 5:**
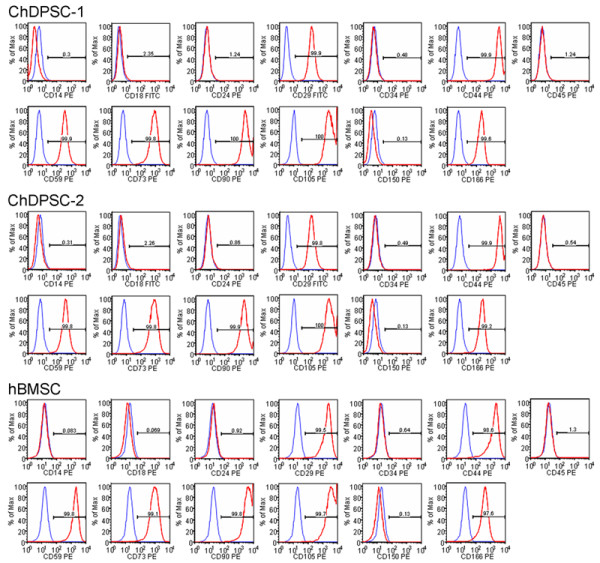
**Cell surface antigen profile in ChDPSCs.** A total of thirteen cell surface antigens were used to characterize the expression profile of ChDPSCs and hBMSCs. Both ChDPSCs and hBMSCs share identical expression profiles on common cell surface antigens. They were all negative for hematopoietic cell surface markers: CD14, CD34, and CD45; yet identical on cell surface markers shared with BMSCs (CD29, CD44, CD59, CD73, CD90, CD105, CD150 and CD166). Furthermore, all cell lines were negative for CD18 and CD24. Blue line: isotype control. Red line: marker of interest.

Besides the expression of stem cell specific transcription factors and a multipotent differentiation capability, the expression profile of a total of 13 cell surface antigens in ChDPSCs and hBMSCs was determined and compared (Figure 5). The cell surface antigen profiles of ChDPSCs and hBMSCs were then compared with published profiles of hBMSCs, hDPSCs and rBMSCs. In both ChDPSC lines, more than 99% of the cells expressed CD29, CD44, CD59, CD73, CD90, CD105, CD150 and CD166, common BMSC markers. However, markers specific for hematopoietic cells, including CD14, CD34, and CD45, were not expressed. Additionally, neither CD18 nor CD24 were expressed in either ChDPSCs or hBMSCs. These results are consistent with previously reported phenotypes of hDPSCs [7], hBMSCs [3], and rBMSCs [42]. In fact, this study was composed of a larger panel of cell surface antigens, revealing an almost identical profile for ChDPSCs and hBMSCs, including hBMSCs run in parallel as well as a published profile of hBMSCs [3]. Based on the expression and distribution pattern of the cell surface antigens, the homogeneity of the ChDPSC population was clearly revealed and was similar to that of the hBMSCs.

## Discussion

The rationale for the establishment of ChDPSCs may be questioned, as DPSCs have already been established from other species, including humans [1,2] and pigs [4,46]. One asset of chimpanzees as a model for humans is the 98% similarity of their genomes [16,23,26,27], making the chimpanzee the closest living relative to humans; it has even been suggested that the chimpanzee should be re-categorized as homo rather than ape. However, the differences between humans and chimpanzees are the most fascinating question that remains unsolved. Due to an almost identical genomic composition, the dissimilarities are most likely not merely a result of the genetic code but due to a difference in regulatory control of specific gene expression that emerged after the evolutionary split of the two species [16,25-27]. Geographic and environmental pressure has further accelerated the divergence of the two species, resulting in the advanced speciation of selective gene function in humans. [27,47,48]. This suggests that the minute differences resulting from the speciation of gene function in humans could be traced only in a genetically close relative, such as the chimpanzee, but not in other more distantly related species, such as monkeys or mice [23].

One example of the distinctness of the chimpanzee from other NHPs is the sequence homology of the brain's *Tau *gene, the key component of filamentous tau deposits in human neurodegenerative diseases [23,25]. The chimpanzee brain tau was 100% identical to that of the human, whereas the gorilla tau was only 99.5% similar, 99% for gibbons, 98% for rhesus monkeys, 88% for mice and 74% for chickens. Additionally, microarray studies on transcriptome in leukocytes and livers suggest that human gene expression patterns are more similar to those of chimpanzees than those of macaques [25]. These studies reflect how the variation in sequence homologies and expression profiles relates to a species' phylogenetic distance from humans. Moreover, the gene expression patterns in the brain demonstrate a unique correlation between chimpanzees and humans that may not be present between humans and other primates [15,23,24,49].

*In vitro *differentiation of DPSCs to a designated lineage or cell type has been difficult because of the lack of knowledge in the area of differentiation control. Although tremendous effort has been invested in developing strategies for *in vitro*-guided differentiation of DPSCs, the underlying regulatory mechanisms remain largely unknown. Spontaneous and guided differentiation has enabled differentiation of hDPSCs to various cell types, including adipocytes [1,2], osteoblasts [1,2], chondrocytes [1,2] and neurons [4]. However, the lack of precise differentiation control and limited homogeneity of the resulting cell types suggest the need for alternate approaches, such as a comparative study using DPSCs derived from a closer species that shares a similar evolutionary path to humans, such as the chimpanzee. A comparative study could accelerate the process of identifying specific regulatory controls that lead to a unique differentiation pathway.

DPSCs are considered to be a potential source of adult stem cells (ASCs) because dental pulp is a vascular connective tissue similar to the mesenchymal tissues found in bone marrow, placenta, muscle, and adipocytes [3,50]. BMSCs and DPSCs have similar gene expression patterns (Figure 4) and cell surface antigen profiles (Figure 5) even though they are two distinct populations of precursor cells [7]. Although RT-PCR confirmed the expression of stem cell specific transcription factors, we were not able to detect at protein level by either immunocytochemistry or western blot, We speculated the expression level of such transcription factors is extremely low in ChDPSCs. Our result also suggested whether the expression of such transcription factors is required for the maintenance of somatic stem cell properties, which is consistent to prior study on the role of Oct-4 in maintaining of somatic stem cell property [51]. Moreover, DPSCs and BMSCs also have similar biofunctions, such as suppressing T-cell functions in the modulation of immuno responses [3]. According to the current classification and criteria of BMSCs [45,52,53], DPSCs are considered to have a lower differentiation potential, with variations in cell surface marker profiles, compared to that of BMSCs [3]. However, the results of our study demonstrated a highly comparable profile between ChDPSCs and hBMSCs (Figure 5; Table 1). Although BMSCs and DPSCs share some common features, they do have different kinetics and differentiation competences [3]. BMSCs are able to develop into osteoblasts, chondrocytes, adipocytes, myelosupportive fibrous-stroma, and other tissue types; whereas DPSCs are capable of developing into osteoblasts with limited evidence of an adipogenic lineage in humans [1-3]. However, we have demonstrated that ChDPSCs have a strong tendency to differentiate into osteoblasts, adipocytes and chondrocytes (Figure 1). It should also be noted that a side-population of DPSCs has been shown to have a more potent differentiation capacity [4]. It has been suggested that DPSCs may represent a subgroup of MSCs at a different stage of differentiation and are therefore committed to a specific function, depending on the microenvironment in which it resides, thus resulting in phenotypic variations [3].

**Table 1:** Comparison of cell surface antigen profile in DPSCs and BMSCs

	ChDPSCs	hBMSCs	rBMSCs^42^	hDPSCs^3^	hBMSCs^45^
CD14*	-	-	-	-	-
CD18	-	-	ND	ND	-
CD24	-	-	ND	ND	-
CD29	+; >99%	+; >99%	+	+	+; >99%
CD34*	-,	-	-	-	-
CD44	+; >99%	+; >99%	ND	+	+; >99%
CD45*	-,	-	-	-	-
CD59	+; >99%	+; >99%	ND	ND	+; 100%
CD73*	+; >99%	+; >99%	ND	ND	+; >99%
CD90*	+; >99%	+; >99%	+	ND	+; >99%
CD105*	+; >99%	+; >99%	ND	ND	+; >99%
CD150	-	-	ND	-	-
CD166	+; >99%	+, >99%	+	+	+, >99%

All values <3% are considered as negative and denoted as "-".

*Important markers for hBMSCs

It is also interesting to note that there was difference in growth pattern between the two ChDPSC lines. We speculated the diverse growth pattern between Ch-DPSC-1 and -2 may be related to the cell type of origin. The two ChDPSCs were derived from the incisor (ChDPSC-1) and the canine (ChDPSC-2) teeth of chimpanzee. The ChDPSC-2 has a more consistent growth rate compared to that of the ChDPSC-1 even they share similar pattern in telomere properties and surface antigen profile. We speculated that canine teeth in species such as chimpanzee continue to grow throughout life and the difference in growth pattern may suggest the difference between teeth type. Thus a comparative study on teeth types merits further investigation.

Compared to bone marrow, dental pulp is easy to access [3], and proliferates at a higher rate (Figure 2). Although the proliferation rate of ChDPSCs decreased after long term passaging (Figure 2), the telomere length was retained (Figure 3). Homogenous populations of DPSCs can be easily isolated from the dental pulp of post-mortem teeth or those extracted for medical reasons throughout life [1-3]. Based on flow cytometry analysis of the expression profile of cell surface antigens, ChDPSCs presented with high homogeneity (Figure 5; Table 1), which suggests a homogenous population of DPSCs could be established efficiently at early passages.

Furthermore, valuable and endangered species such as chimpanzees, pandas, gorillas, and Siberian tigers, are mostly housed in sanctuaries, research institutions or zoos. Although these species are relatively uncommon in biological studies, they may hold the key to understanding the role of evolution in cell specification and differentiation control. Unfortunately, it is difficult to collect bone marrow, gametes, and other viable tissues from these unique species for stem cell isolation; and it is practically impossible to collect embryonic stem cells (ESCs). Primary cultures from the muscle, the liver, and the brain could be established when tissue is retrieved at necropsy. Unfortunately, the isolation of adult stem cells (ASCs) may not be a practical approach, as most sanctuaries and zoos are not equipped with the tools and skills for emergency recovery of bone marrow or brain tissues for stem cell isolation. Specific preservation and retrieval procedures are often required, and isolation is usually performed immediately upon retrieval in order to have a high recovery yield. Additionally, most ASCs are relatively low in abundance and often difficult to isolate. The exception is the isolation of BMSCs from bone marrow and cord blood where efficient isolation procedures have been established. Based on these reasons, the isolation of ASCs is not possible for most of these species, unless arrangements have been well planned in advance. On the other hand, the isolation of DPSCs from dental pulp is simple and has a high success rate. Most necropsy facilities have the tools for teeth retrieval and no special preservation or handling procedures are required. Teeth could be retrieved, preserved in saline, kept at 4°C, and would then be ready for shipment. In fact, teeth preserved overnight at 4°C have a comparable isolation efficiency compared with fresh dental pulp from monkeys (unpublished data). Therefore, it is possible to recover DPSCs from valuable and endangered species with no geographic limitations. Stem cells from such species are invaluable research materials for studying differentiation control in species with unique habitats and positions on the phylogeny tree.

## Conclusion

In conclusion, DPSCs are easy to establish from the dental pulp, capable of self-renewal, and differentiate to multiple lineages. Although DPSCs are primarily considered to have applications in paradontology, implantology, and calcified tissue reconstruction [3,4,6,53,54], clinical application of DPSCs should not be limited to hard tissue engineering. It seems that DPSCs have a similar, if not identical, potential to BMSCs [53], and therefore more diversified clinical applications should be investigated. Most importantly, ChDPSCs are valuable as a comparative model in ASC research, which may lead to insights into the understanding of stem cell properties and differentiation control in higher primates, such as humans.

## Authors' contributions

PHC: ChDPSC culture and characterization. BS: hBMSCs culture and preparation for FACs. DF and CI: FACs analysis. AHCH: experimental design. AWSC: ChDPSC isolation, experimental design and manuscript preparation. All authors read and approved the final manuscript.
